# Validation of the Neurological Fatigue Index for stroke (NFI-Stroke)

**DOI:** 10.1186/1477-7525-10-51

**Published:** 2012-05-15

**Authors:** Roger J Mills, Julie F Pallant, Maria Koufali, Anil Sharma, Suzanne Day, Alan Tennant, Carolyn A Young

**Affiliations:** 1 The Walton Centre for Neurology and Neurosurgery, Liverpool, UK; 2 School of Rural Health, University of Melbourne, 49 Graham St, Shepparton, VIC, 3630, Australia; 3 Stroke Team for Audit & Research, University Hospitals Aintree, Liverpool, UK; 4 Department of Rehabilitation Medicine, University of Leeds, D Floor, Martin Wing, Leeds General Infirmary, Gt. George Street, Leeds, LS1 3EX, UK; 5 Department of Neurology, Royal Preston Hospital, Sharoe Green Lane Fulwood, Preston, PR2 9HT, UK

**Keywords:** Stroke, Multiple sclerosis, Fatigue, Scale, Rasch analysis

## Abstract

**Background:**

Fatigue is a common symptom in Stroke. Several self-report scales are available to measure this debilitating symptom but concern has been expressed about their construct validity.

**Objective:**

To examine the reliability and validity of a recently developed scale for multiple sclerosis (MS) fatigue, the Neurological Fatigue Index (NFI-MS), in a sample of stroke patients.

**Method:**

Six patients with stroke participated in qualitative interviews which were analysed and the themes compared for equivalence to those derived from existing data on MS fatigue. 999 questionnaire packs were sent to those with a stroke within the past four years. Data from the four subscales, and the Summary scale of the NFI-MS were fitted to the Rasch measurement model.

**Results:**

Themes identified by stroke patients were consistent with those identified by those with MS. 282 questionnaires were returned and respondents had a mean age of 67.3 years; 62% were male, and were on average 17.2 (SD 11.4, range 2–50) months post stroke. The Physical, Cognitive and Summary scales all showed good fit to the model, were unidimensional, and free of differential item functioning by age, sex and time. The sleep scales failed to show adequate fit in their current format.

**Conclusion:**

Post stroke fatigue appears to be represented by a combination of physical and cognitive components, confirmed by both qualitative and quantitative processes. The NFI-Stroke, comprising a Physical and Cognitive subscale, and a 10-item Summary scale, meets the strictest measurement requirements. Fit to the Rasch model allows conversion of ordinal raw scores to a linear metric.

## Introduction

Fatigue is a common symptom in stroke [1,2]. It can be considered to be ‘a feeling of early exhaustion, weariness and aversion to effort’ [3], or a ‘lack of energy with an increased need to rest’ [4]. The extent of fatigue has been shown to increase with stroke severity[5]. It can have a considerable impact upon lifestyle and has, for example, been shown to be an independent predictor for the need to move into an institutional setting post-stroke [6]. It has also been shown to have association with depression, and sleeping problems [7].

Given the importance of post-stroke fatigue, several fatigue scales have been used to ascertain the extent of fatigue experienced. Examples include the Fatigue Assessment Scale [8]; the Multidimensional Fatigue Inventory (MFI-20) [9] the Fatigue Severity Scale [10]; and the Brief Fatigue Inventory [11]. A recent review of some of these scales suggested varying levels of reliability and validity, with no one scale showing satisfactory results across all psychometric quality indicators [12]. Consequently it has been argued that a more exact definition of fatigue is needed, and then more valid scales or other technical instruments to quantify fatigue [13]. One such scale, the Neurological Fatigue Index (NFI-MS), was developed from theory and the experiences of those with multiple sclerosis [14,15].

This current paper sets out to examine if the thematic structure relating to fatigue which emerged from that MS study is also consistent with those who have experienced a stroke, and to test the reliability and validity of the NFI-MS in stroke (any valid subscales would then also be known as NFI-Stroke).

## Methods

The study had approval from the local research ethics committee (Sefton EC115.03 and 05/Q1501/24). All subjects received written information on the study and gave written informed consent prior to participation.

### Sample and materials

#### Qualitative construct validation

Semi-structured interviews with stroke patients were used to identify the features which defined the concept of fatigue. Subjects with radiologically confirmed stroke were recruited, non-purposively, to undergo a semi-structured interview, as they attended the out patient clinic in the Department of Medicine for the Elderly at University Hospitals Aintree, Liverpool and the Neurology Rehabilitation Unit, Walton Centre for Neurology and Neurosurgery, Liverpool, UK. Patients were excluded if they either had marked impairment of communication or they had another neurological condition. The same interviewer (SD), blinded to the results of the NFI-MS, was used throughout and the face-to-face interviews audio-taped and later transcribed. The ‘framework approach’ [16] was used for the qualitative analysis of the interview transcripts. This part of the study mirrored the qualitative work which had already been undertaken in forty patients with multiple sclerosis and which formed the basis of the NFI-MS; the method is described in detail elsewhere [14].

The post-stroke qualitative analysis was examined for thematic equivalence against the MS data. It was decided *a priori* that if post-stroke fatigue was found to be qualitatively identical to MS fatigue, then the existing NFI-MS item set would be used, otherwise further interviews would be undertaken, and new items would be generated to represent any stroke-specific features of fatigue.

#### NFI-MS

The NFI-MS consists of 23 items in four subscales of Physical (8 items), Cognitive (4 items), Relief by diurnal sleep or rest (6 items) and Abnormal nocturnal sleep and sleepiness (5 items). A 10-item Summary Scale derived from physical and cognitive items is also available. Wording of the scales is both simple and concise; the use of the word ‘fatigue’ was deliberately avoided because of its associated semantic ambiguities. All items are worded in such a way as to be scored in the same direction. Each item has a four point, Likert response option [17] with headings of ‘strongly disagree’, ‘disagree’, ‘agree’ and ‘strongly agree’, which progress in the natural reading direction (*i.e.* left to right), and are scored 0, 1, 2, 3. There is a single sentence instruction at the start of the scale asking respondents to consider their experience over the previous four weeks.

#### Data collection

A pack containing the NFI-MS, other measures and questions on demographics and basic disease information, was mailed to a random cross-section of stroke patients identified from the Aintree Stroke Register held at the University Hospital Aintree, Liverpool, UK. All patients had one or more radiologically confirmed stroke(s) in the previous 50 months. The type of stroke (ischaemic or intracerebral haemorrhage) was known from the stroke register but the Oxfordshire Community Stroke Project subtype [18] was not available. There were 4,276 patients in the registry with the clinical and demographic details having been obtained prospectively during admission to hospital.

The Fatigue Severity Scale (FSS) [10], a nine item scale with a seven-point response option, was co-administered as a comparator measure. The FSS is the most frequently used scale in stroke fatigue [19]. In addition, a 10 cm, modified, vertical, visual analogue scale (VAS) with anchors of ‘lively and alert’ and ‘absolutely no energy to do anything at all’ was co-administered; similar vertical visual analogue scales have been widely used [20,21].

Estimation of disability was made by administration of the Stroke Impact Scale-16 (SIS) [22]. This is a short form version of the Stroke Impact Scale v2.0 [21]. with items based on mobility and activities of daily living, each having a 5-point Likert response; minimum possible score was zero (no meaningful disability) and the maximum was 64. Hemianopia and visual neglect, which might interfere with completion the response option, were assessed by copy of a clock face.

In total, 999 people received the pack. Retesting was performed at 2 to 4 weeks on the first 80 respondents to the main mailout; estimates of the level of fatigue would be correlated between initial and retest time points accepting a Spearman’s rho of ≥0.7. Invariance of mean person estimates at each time point would be confirmed by paired *t*-test. For analysis of the Rasch fit criteria, only the initial time point data and not the retest data, were included. Data were transcribed to a computer database (transcription error based on checking a random 10% sample was <0.1%, missing data accounted for 3.8% of total).

If the response to the mailout was less than 50%, then non-response bias would be assessed by *t*-test or chi square comparison for: age at onset of most recent stroke, sex, previous stroke, previous transient ischaemic attack (TIA) and stroke type. This would be performed to exclude any gross bias in the responders, but it must be stressed that population representativeness is *not* a requirement for Rasch analysis, but rather a wide range of person ‘ability’ (in this case levels of fatigue) is needed [23].

#### Quantitative psychometric analysis

##### Measurement and the rasch model

The internal construct validity of the NFI-MS in stroke was examined by fit of data to the Rasch measurement model [24]. Full details of the process of Rasch analysis are given elsewhere [25,26]. Briefly, the process is concerned with whether or not the data meets the model expectations, and provides an assessment of the suitability of the response scale, the fit of individual items, differential item functioning, and the dimensionality and targeting of the scale as a whole.

In summary, fit of data to the Rasch model was deemed acceptable if the following criteria were fulfilled:

1) ordered item category thresholds;

2) both total chi-square probability and individual item chi-square probability values non-significant (5% alpha with Bonferroni correction for the number of items);

3) individual item fit residual, by convention, within ±2.5;

4) mean and SD of both item fit residual and person fit residual approaching 0 and 1 respectively;

5) person-item separation index (PSI) (reliability) greater than 0.70 for group use and 0.85 for individual use;

6) ANOVA probability for differential item functioning (DIF) non-significant (5% alpha with Bonferroni correction) for the following factors: sex, age and whether had help (as a scribe) completing the scale, as well as time point (*i.e.* initial and retest). This is undertaken with a two way ANOVA with class interval (grouped level of fatigue) and the external factor (*e.g.* age) as main effects. Uniform DIF is then for the main effect of gender (and there is another for class interval) and non-uniform DIF is the interaction between class interval and (*e.g.* age).

7) Unidimensionality by independent *t*-test at the person level showing less than 5% of tests to be significant (or the lower bound of the binomial confidence interval to overlap 5%, where required) [27,28].

8) Pearson correlation coefficients between item residuals less than 0.3 (local independence).

For Rasch analysis, a sample size of 243 will provide accurate estimates of item and person locations irrespective of the scale targeting [29]. The Rasch analysis was performed using the RUMM 2020 computer software (http://www.rummlab.com). The unrestricted (partial credit) Rasch polytomous model was used with a conditional pair-wise parameter estimation [30]. Failure of items to fit Rasch model expectations led to an iterative procedure using techniques for collapsing response categories, item deletion, and adjusting for DIF where necessary.

If data from a scale fit the Rasch model, then the summed ordinal raw scores can be considered to be sufficient to determine the level of fatigue in an individual. However, calculation of change scores can only be done with interval level data and so a conversion table of the raw ordinal score to the interval level metric, for any resultant scale, would be provided.

#### External comparison

Comparisons of the person locations from the final scale to the summed raw scores of the FSS, VAS, and SIS were made by Spearman correlation (assuming the raw scores were non-parametric), with the expectation that these would be mild to moderate (*i.e.* rho between 0.3 and 0.7) in each case [31].

## Results

### Qualitative analysis

Five of the six patients recruited for the qualitative interviews were female. The mean age of participants was 51.3 years (SD 13.4, range 34–68), the mean duration since last stroke was 22 months (SD 39.3 range 3–108). The interview data were analysed in the context of a ‘framework’ of standard symptom description. Complete thematic equivalence of the stroke data was observed when compared to the existing MS data. In other words, no new features, specific to post-stroke fatigue, were identified when compared to MS fatigue (Table 1).

**Table 1:** Some examples of the features of post-stroke fatigue, as described by patients, grouped according to the thematic framework derived from MS

**MS Framework**	**Stroke quotations**
Subjective experience	*Basically just tiredness to the point where you’re worn out. Tired. Done in.*
	*Tiredness all the time… Its just tiredness, constant tiredness*
	*Shattered. No energy. Whacked out. Weary.*
motor	*It just takes it out your body. You just want to lie down and you’re drained.*
	*If I do anything, you know, anything physical. Or go to the shops. Really shatters me.*
cognitive	*I’m still not reading…I can’t concentrate on it.*
	*It feels, sort, of, my eyes start going cos I’ve got to concentrate on the story.*
motivation, energy and need to rest	*If I know I’ve got something to do, I’m quite happy to get on and do it. But if I know its not that day then I’m tired and I can’t be bothered doing it…*
	*But my fatigue is, when I get home here in my bedroom, I sort of give in then*
	*ll take my son to school, get back in the car and go home and I’ll go straight back to bed for a few hours. But if I’m busy of a morning, I’ll go to bed at lunchtime*
Sleep and behavioural response	*I’m really tired and just want to go to bed and sleep and not bother with anything*
	*Yes, well then the tiredness takes over. Basically I want to stay sitting down then and I’m weary*
	*Sometimes you feel like when you do get up you’re tired more’*

### Quantitative analysis

#### Subjects and non-responder analysis

284 packs were returned and two were discarded because of evidence of substantial visual field defect. This gave a 28.2% (282/999) response, sufficient for the Rasch analyses. The demographic details and disease characteristics are given in Table 2. Indication of the functional consequence of stroke is also given. The median SIS score was 17 which equates to a Modified Rankin Score [32] of 2 (slight disability) [22], but a full range of disability was observed, for instance subjects with severe disability of both upper and lower limb function were represented (see Table 2 for frequencies). The VAS fatigue scores were normally distributed (skewness -0.25, kurtosis -0.39) with median and modal values both of 5 cm. The distribution of the FSS revealed a substantial ceiling effect of 7.1%. Histograms of the VAS, FSS and SIS can be found in the supplemental material.

**Table 2:** Characteristics of subjects both completing the pack and non-responders

	**responders**	**non-responders**
n	282	717
mean age at questionnaire completion (SD, range)	67.3 (13.4, 18–95)	–
mean age at onset of last stroke (SD, range)	66.5 (12.4, 18–93)	70.9 (12.8, 16–102)
male (%)	61.3	45.8
mean months post stroke (SD, range)	17.2 (11.4, 2–50)	–
previous stroke (%)	9.6	21.1
previous TIA (%)	11.6	16.6
ischaemic stroke (%)	78.7	75.3
working (%)	16.5	–
median Stroke Impact Scale score (range)	17 (0–64)	–
very difficult or unable to climb one flight of stairs (%)	25.9	–
very difficult or unable to dress top half of body (%)	10.1	–
very difficult or unable to control bladder (%)	8.3	–
very difficult or unable to transfer from bed to chair (%)	5.2	–

Ages are in years. SD-standard deviation.

76 records were available for the retest analysis.

The non-responders, when compared to the responders, were slightly older with a mean age difference of 4.4 year (95% CI 2.6–6.2, p < 0.001). A greater proportion were female (53% vs. 38%, p < 0.001) and had suffered more than one stroke (21% vs. 10%, p < 0.001) but there was no difference in previous TIA (p = 0.062) or type of stroke (p = 0.334). None of the differences were considered to be extreme or confound the validity of the Rasch analysis.

#### Rasch analysis

**Figure 1:**
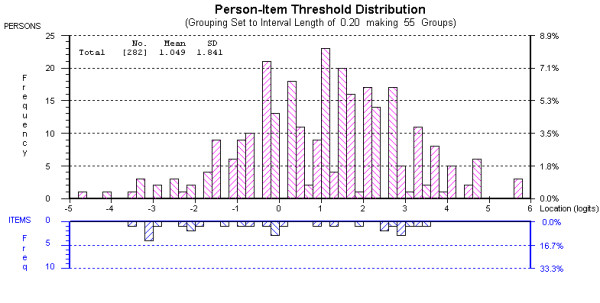
Person Item Distribution of the NFI-Stroke Summary Scale.

Data from the four individual subscales, and the summary scale of the NFI were then fitted to the Rasch measurement model. The findings, related to the analysis of each domain, are given in Table 3. All thresholds were ordered in all subscales. Data from the Physical subscale satisfied Rasch model expectations, were unidimensional, free of DIF and local dependency (Table 3, Analysis 1). Likewise, data from the Cognitive subscale also satisfied model expectations and were free of DIF and local dependency, although the reliability (PSI) was only consistent with group use and the summary item residual standard deviation was a little high, and one item (coordination gets worse) had a slightly high negative residual at -2.75 (Table 3, Analysis 2). The summary scale also satisfied model expectations and was unidimensional and free of DIF and local dependency (Table 3, Analysis 3). Overall, respondents had a slightly higher level of fatigue (1.04 logits) than the average of the scale (0.0 logits) (Figure 1). These three scales could now be called NFI-Stroke.

**Table 3:** Summary Fit Statistics for Rasch analyses

**Analysis Number**	**Analysis Name**	**Item Residual**		**Person Residual**		**Chi-Square**		**PSI**	**Unidimensional**
									***t*-test**
									**(CI)**
		***Mean***	***SD***	***Mean***	***SD***	***Value***	***p***		
1	Physical (8 items)	-0.379	1.011	-0.547	1.415	21.4	0.922	0.89	5.98%
									(3.2-8.8)
2	Cognitive (4 items)	-0.043	1.977	-0.597	1.224	18.9	0.092	0.78	3.29%
									(0.4-6.2)
3	Summary (10 items)	-0.357	1.156	-0.622	1.551	52.8	0.085	0.89	5.0%
									(2.2-7.8)
4	Diurnal – Initial (6 items)	-0.423	1.862	-0.683	1.351	50.5	0.001	0.70	7.72%
									(5.1-10.4)
5	Diurnal – Final (5 items)	-0.842	1.783	-0.648	1.174	18.9	0.219	0.69	5.56%
									(2.8-8.3)
6	Nocturnal Sleep (5 items)	0.229	1.451	-0.563	1.501	31.5	0.008	0.69	4.22%
									(1.4-7.0)
	***Acceptable Values***	***0***	***<1.4***	***0***	***<1.4***		***>0.05***^a^	***>0.85***	***< 5.0% (Lower CI)***

^a^ Bonferroni adjusted alpha level.

The sleep scales were more problematic. The Diurnal sleep scale failed to meet model expectations (Table 3, Analysis 4). The item ‘I try to get everything done in the morning’ showed misfit, with a significant Chi-Square statistic, and the item set displayed multidimensionality (7.72%; CI 5.1-10.4%). Removal of this item improved fit, and resulted in a unidimensional scale (Table 3, Analysis 5). However, the item residual standard deviation, at 1.783, indicated some continuing misfit caused by another item with a high negative fit residual, indicating redundancy. The Nocturnal sleep subscale failed to fit the model (Table 3, Analysis 6), and no solution could be found.

#### External construct validity

Comparison of the person locations from the Physical, Cognitive and Summary Scales to the FSS gave Spearman correlation coefficients of 0.604, 0.509 and 0.622 respectively. Likewise, to the VAS, 0.556, 0.385 and 0.534 respectively, and to the SIS of 0.615, 0.532 and 0.628 respectively.

#### Test-retest

Test-retest correlation coefficients of the Physical, Cognitive and Summary Scales were 0.903, 0.786 and 0.896 respectively. There were no significant differences in the mean scores at the two time points. Analysis of DIF by time showed that the Physical, Cognitive and Summary scales were invariant between the initial and retest time points.

#### Raw score to interval scale conversion

Given fit to the Rasch model of the Physical, Cognitive and Summary Scales, a straightforward conversion is available between the raw score for each scale, and the interval scale estimate of the latent trait of fatigue provided by the model (Table 4). This can be used when data are complete.

**Table 4:** Raw score to interval scale conversion table for the scales

**Raw**	**Summary**	**Physical**	**Cognitive**
**Score**	**Scale**	**Scale**	**Scale**
0	0.00	0.00	0.00
1	2.34	1.97	1.46
2	4.05	3.43	2.69
3	5.29	4.51	3.73
4	6.31	5.42	4.70
5	7.22	6.24	5.60
6	8.05	7.00	6.43
7	8.83	7.73	7.21
8	9.59	8.45	7.98
9	10.32	9.16	8.78
10	11.05	9.88	9.65
11	11.77	10.61	10.71
12	12.49	11.35	12.00
13	13.22	12.13	
14	13.96	12.94	
15	14.70	13.78	
16	15.46	14.65	
17	16.24	15.53	
18	17.03	16.41	
19	17.83	17.29	
20	18.64	18.21	
21	19.44	19.21	
22	20.25	20.38	
23	21.06	21.93	
24	21.89	24.00	
25	22.75		
26	23.67		
27	24.71		
28	25.95		
29	27.66		
30	30.00		

N.B. The conversions only remain valid if there are no missing data. The transformation can only occur when data are complete because, for example, a score of say 6 from complete data is not the same as a score of 6 from incomplete data. The latter is likely to represent a higher level of the attribute being measured.

## Discussion

Qualitative construct validation in adapting scales to a different diagnosis is novel. By testing the construct equivalence from the current sample against the original qualitative analysis from people with MS, the manifestation of post-stroke fatigue appeared to be qualitatively similar to that of MS fatigue, including, for example, features associated with physical and cognitive aspects [14]. The NFI-Stroke reflects these components, and provides a simple scale of fatigue that satisfies the strictest measurement standards, supporting the internal construct validity of the scale. The substantive correlations (>0.5) with the comparator measures provide strong evidence of the external validity of the scale, with slightly stronger correlations with the physically orientated domains of the FSS and SIS. The lower correlation of the cognitive scale with the VAS possibly reflects the choice of anchors for the latter which were necessarily concise and may not have conveyed the nuances of cognitive fatigue.

The qualitative similarity between post-stroke fatigue and MS fatigue and indeed the facility with which fatigue in the two conditions could be measured on a common metric is notable given the obvious differences in pathophysiology between the two diseases. This paradox may be a potentially important starting point for future pathophysiological enquiry.

The raw score from the NFI-Stroke components is a sufficient statistic such that a simple summed score can provide an ordinal estimate of the persons (component) level of fatigue. It also can provide a straightforward ordinal to interval scale transformation, courtesy of a special property of the Rasch model [24,33].

There were a number of limitations to the study. For example, the sample was restricted to those with a disease duration of 4 years from their most recent stroke; this was mainly determined by the age of the stroke register from which the sample was drawn. Fatigue seems to increase in the first twelve months [34] following stroke but is known to persist for more than two years [6] and so four years was felt to be an adequate disease duration for the current sample.

Subjects had to be cognitively able to interpret and respond to the scale. Further validation of the NFI-Stroke might involve clinician administration to patients with cognitive deficits.

The qualitative stroke sample was predominantly female and it could be argued that equivalence of the thematic structure to MS was confounded by sex bias. However, no sex differences were found in the MS qualitative analysis [14] and in the current Rasch analysis, all items were free from DIF by sex.

The non-response level of the study was also high and older patients with multiple strokes appeared to have been underrepresented. Respondents with low levels of disability were well represented. However some respondents had very high SIS scores suggesting that those with higher disability were not wholly excluded. In addition, the VAS scores were normally distributed suggesting those with extreme levels of fatigue (both low and high) were captured. Nevertheless, representativeness is not a requirement for Rasch analysis as, for example, item difficulty estimates are independent of the distribution of the sample of persons [23]. The fact that the Summary Scale was adequately targeted to the sample, and that the sample covered a wide range of those with low to high fatigue is more important with respect to the construction of a measure than the sample’s representative nature. It should be remembered that patients were being presented with a whole pack of different scales and demographic questions which may have contributed to inflation of the non-response rate. The NFI-Stroke is a brief scale with straightforward and concise items. This format is in-keeping with other self-report scales used in neurologic disease, including stroke, and so there was minimal concern that non-response was due to some deterrent intrinsic to the structure of the NFI-Stroke.

There was one item in the Cognitive scale (coordination gets worse) with a slightly high negative fit residual which indicated a degree of redundancy and accounted for the inflated overall item residual standard deviation. The scale was not discarded because all other fit statistics were acceptable and the retention of a comparable cognitive fatigue scale between stroke and MS was felt to be desirable. Additionally, the same item had satisfactory fit within the Summary scale, albeit with the lowest chi square probability.

The sleep scales were found to be less than optimal. This was also the case in the context of MS, and thus remains a challenge for measurement. Both sets of diagnostic-specific qualitative analysis supported the importance of sleep (or its disturbance) and therefore further work is required to develop the NFI sleep scales for these populations. Whether, or not, relief by diurnal sleep or rest is adaptive or consequent, remains to be determined. It is possible that diurnal sleep represents an inherent part of the pathophysiology of fatigue, but it could also be a secondary behavioural response. The Diurnal sleep scale overall fit statistics only showed a high item residual standard deviation, comparable to the Cognitive scale. However, because, even after some modification, there was persistent individual item misfit the scale was discarded. Unlike MS, the Abnormalities of Nocturnal Sleep scale could not be resolved for the current diagnostic group.

Given the interval scaling of the NFI-Stroke, the potential now exists to model the antecedent and consequent factors associated with fatigue, and the associations within the broader biopsychosocial model, using path analysis or other appropriate multivariate techniques.

## Conclusion

The NFI-Stroke provides a brief (12 item) and easy-to-use tool for measurement of a clearly defined concept of fatigue. The scale satisfies strict Rasch model measurement requirements and, as a result, interval level scaling is available for when change scores need to be calculated. The scales have specific validation for stroke and can be used on patients of, amongst other factors, any age, or sex.

It is suggested that the scale would be useful in both a clinical setting and as an outcome measure in clinical trials. The NFI-Stroke is free for use by all state-funded healthcare organisations and not-for-profit agencies, and can be obtained, after appropriate registration, from http://www.leeds.ac.uk/medicine/rehabmed/psychometric/Scales1.htm or by contact of the authors.

## Competing Interests

The authors declare that they have no competing interests.

## Author’s Contributions

RJM, CAY, MK, SD and AS contributed to the design, implementation, and analysis of the study. JFP and AT contributed to the analysis of the study. All authors contributed to the writing of the manuscript, and all approved the final version.

## Supplementary Material

Additional file 1 Histograms showing the distribution of the visual analogue scale (VAS), Fatigue Severity Scale (FSS) and Stroke impact Scale (SIS). Click here for file
