# Supplementary material for: Validation of the Neurological Fatigue Index for stroke (NFI-Stroke)
Source: Health Qual Life Outcomes. 2012 May 15;10:51. doi: 10.1186/1477-7525-10-51 (PMC3485136; doi:10.1186/1477-7525-10-51)

Supplementary material

Histograms showing the distribution of the visual analogue scale (VAS), Fatigue Severity Scale (FSS) and Stroke impact Scale (SIS)


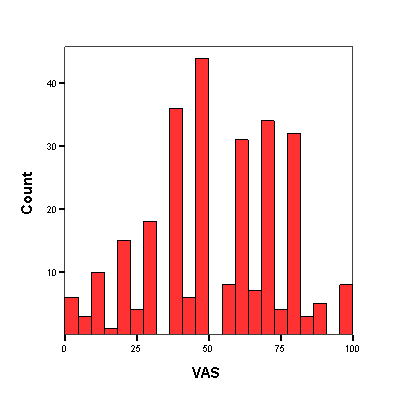


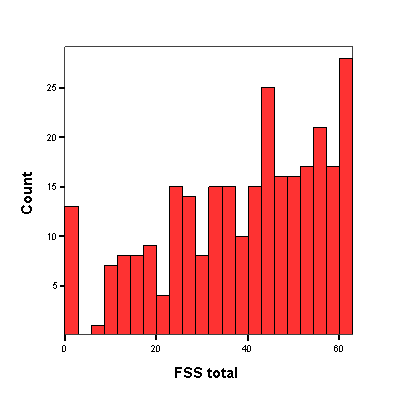


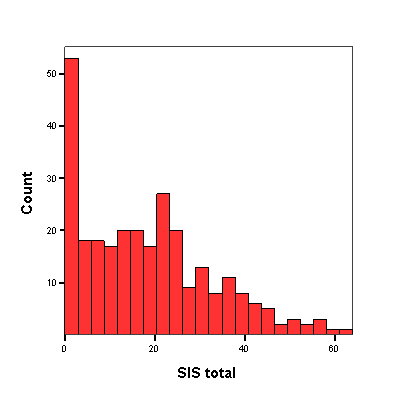

Supplement: Additional file 1 — Histograms showing the distribution of the visual analogue scale (VAS), Fatigue Severity Scale (FSS) and Stroke impact Scale (SIS). [file 1477-7525-10-51-S1.doc]
